# A Mathematical Model of T Lymphocyte Calcium Dynamics Derived from Single Transmembrane Protein Properties

**DOI:** 10.3389/fimmu.2013.00277

**Published:** 2013-09-18

**Authors:** Christine Schmeitz, Esteban Abelardo Hernandez-Vargas, Ralf Fliegert, Andreas H. Guse, Michael Meyer-Hermann

**Affiliations:** ^1^Department of Systems Immunology, Helmholtz Centre for Infection Research, Braunschweig, Germany; ^2^The Calcium Signalling Group, Department of Biochemistry and Molecular Cell Biology, University Medical Center Hamburg-Eppendorf, Hamburg, Germany; ^3^Department of Life Sciences, Technische Universität Braunschweig, Braunschweig, Germany

**Keywords:** T lymphocytes, calcium dynamics, mathematical model, CRAC, endoplasmic reticulum

## Abstract

Fate decision processes of T lymphocytes are crucial for health and disease. Whether a T lymphocyte is activated, divides, gets anergic, or initiates apoptosis depends on extracellular triggers and intracellular signaling. Free cytosolic calcium dynamics plays an important role in this context. The relative contributions of store-derived calcium entry and calcium entry from extracellular space to T lymphocyte activation are still a matter of debate. Here we develop a quantitative mathematical model of T lymphocyte calcium dynamics in order to establish a tool which allows to disentangle cause-effect relationships between ion fluxes and observed calcium time courses. The model is based on single transmembrane protein characteristics which have been determined in independent experiments. This reduces the number of unknown parameters in the model to a minimum and ensures the predictive power of the model. Simulation results are subsequently used for an analysis of whole cell calcium dynamics measured under various experimental conditions. The model accounts for a variety of these conditions, which supports the suitability of the modeling approach. The simulation results suggest a model in which calcium dynamics dominantly relies on the opening of channels in calcium stores while calcium entry through calcium-release activated channels (CRAC) is more associated with the maintenance of the T lymphocyte calcium levels and prevents the cell from calcium depletion. Our findings indicate that CRAC guarantees a long-term stable calcium level which is required for cell survival and sustained calcium enhancement.

## 1 Introduction

The dynamics of the free cytosolic calcium concentration upon stimulation of T lymphocytes (TCs) is crucial for TC activation and fate decision processes. While it is clear that calcium rises upon stimulation of the TC receptor (TCR), the calcium pattern associated with different fates of TCs has not been deciphered (1–4). However, it is likely that the calcium signal is correlated with the later fate of the activated TC, i.e., anergy, division, acquisition of the regulatory phenotype or apoptosis (3, 5, 6). Dysregulation in TC calcium signaling has been linked to inflammatory and autoimmune diseases as well as to allograft rejection (2). A relevant player in calcium dynamics is the calcium-release activated channel (CRAC) which is located in the plasma-membrane and activated by calcium depletion in the intracellular calcium stores like the endoplasmic reticulum (ER). As ion-gating transmembrane proteins in the plasma-membrane (PM) are possible targets of drug applications in the context of various clinical settings (2, 7, 8), insight into the specific calcium dynamics is essential for an efficient control of TC behavior.

The relative contribution of ER-derived calcium entry versus CRAC to the calcium signal following TC stimulation is a matter of ongoing debate (9–12). While a considerable number of scientists argue for CRAC being the major player of TC calcium dynamics (12, 13), others argue for a dominant role of calcium-induced calcium-release (CICR) (10, 14, 15) or for a dominant role of second messenger-induced calcium-release from ER (16, 17). All three contributions are required for a functional TC calcium signal, however, the sequence of the contributions might be essential. Second messenger-induced activity appears as a very early signal (18), which might act as triggering event for CICR and subsequent CRAC activation (2, 19–21). Quantitative analysis of the components of calcium signaling during TC activation is essential for the development of strategies for an efficient control of TC responses. A mathematical analysis of the calcium dynamics in a model including calcium stores and CRAC may shed light on the relation and relevance of both calcium sources (12, 22). This is the major motivation for the present work.

T lymphocytes are non-excitable cells in the sense that they do not exhibit bursts like pancreatic beta-cells, spikes like neurons, or comparable fast dynamics (23–25) even though single cell measurements detected calcium oscillations (10, 19, 26). The non-excitability of TCs might have prevented a larger interest of mathematical modelers in lymphocyte calcium dynamics. The few existing models (22, 27–29) mostly focused on modeling of CRAC-channel dynamics or a special part of the store-operated-calcium-entry signaling pathway (27) and its contribution to intracellular calcium dynamics. Also the dependence of ORAI1 assembly to a tetrameric CRAC on calcium oscillations was considered (28). In one approach a spatial resolution of CRAC currents and of the calcium dynamics in TCs was considered in the context of immunological synapse formation (22). Two different models for inositol 1,4,5-tris-phosphate-receptor (IP3R) activity were compared and it was shown that they differ in their impact on TC calcium dynamics (27), a result that will be used in the present model as well. The plasma-membrane calcium-ATPase (PMCA) was modeled in Jurkat TCs and a reversible modulation of PMCA activity was postulated (12), which is a further topic addressed in this investigation.

The aforementioned theoretical studies on TC calcium dynamics were all based on whole cell current models. In the context of excitable cells we have shown that it is possible to derive the whole cell currents from the single transmembrane properties (30). To achieve this goal, specific quantitative measurements of protein activation, inactivation, dependencies and conductance were used. The big advantage of this approach is that most parameters of the model are determined by independent experiments, which increases the predictive power of the model. The present paper applies this strategy to calcium dynamics of TCs. The model also includes the dynamics of transmembrane channel expression kinetics in order to represent CRAC recruitment upon calcium store depletion. The mathematical model was validated using dynamic calcium data measured under specific experimental conditions. The validated model allowed to reassess the relative role of store-derived and CRAC-mediated calcium entry on a quantitative basis. A new role of the CRAC-channel is postulated, which is associated with maintenance of TC calcium levels rather than TC activation.

## 2 Materials and Methods

**Figure 1:**
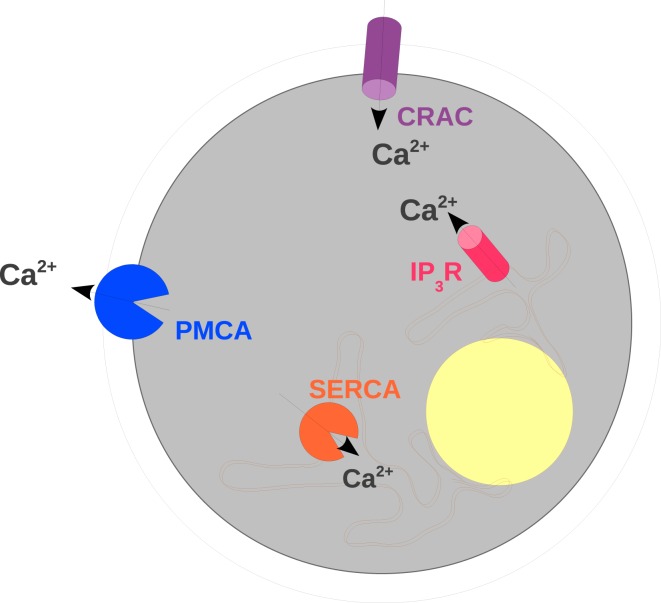
**Scheme of the transmembrane proteins included in the mathematical model**. The elements of a TC considered in the mathematical model are shown with particular emphasis on calcium-conducting transmembrane proteins. The outer border of the cell represents the PM. In the PM CRAC and PMCA are located which induce calcium influx and extrusion, respectively. The intracellular organelle around the nucleus (yellow) depicts the ER. The membrane of the ER contains IP3R and SERCA (sarco/endoplasmic reticulum calcium-ATPase) which control the exchange of calcium between the ER and the cytosol.

The modeling framework is presented in this section. Three compartments, extracellular space (ES), cytosol, and ER are distinguished, each being represented by ordinary differential equations. The nucleus is only included as an object which reduces intracellular space (Section 2.4). The compartments are encased by the PM and the membrane of the ER. Both membranes contain transmembrane proteins (Figure 1), which allow for a flow of ions from one compartment to the other. The surface densities and the properties of these proteins in terms of conductance and control parameters determine the resting and activated states of the TC. While the protein properties are derived from measured single protein data, which are independent of the whole cell calcium experiments used for validation, the protein densities are in parts derived from steady state conditions (Section 2.9) and in parts determined by fitting to whole cell calcium dynamics (Section 2.10).

In the following, the equations for calcium dynamics, calcium-buffering, and for the second messenger d-myo-inositol-1,4,5-tris-phosphate (IP3) are introduced. The details of the compartment sizes and the surface between the compartments are explained in Section 2.4. Particular emphasis is put on the geometry of the Jurkat TC, the specific cell line, which is used in the subsequently described experiments. The single transmembrane protein characteristics will be described and the corresponding mathematical models introduced. Wherever possible we implemented data from experiments performed with Jurkat TCs. Finally, all model pieces are merged to the proposed model of TC calcium dynamics in Section 2.10. This includes the determination of the remaining unknown parameters.

### 2.1 Calcium dynamics and buffering

Two major players determine exchange of calcium through the PM (Figure 1): PMCAs actively transport calcium from the cytosol into ES (12, 31), while CRAC-channels allow for a passive electrochemical current of calcium ions into the cell (21, 32, 33). The density of active CRAC-channels ρ_CRAC_ in the PM is increased in dependence on ER calcium (*C*_ER_) depletion (34). The free cytosolic calcium concentration (*C*) is further affected by modulations of the calcium flow between cytosol and ER (Figure 1). Sarco/endoplasmic reticulum calcium-ATPase (SERCA) transports calcium ions from the cytosol into the ER (10, 35) and by this maintains a chemical gradient of calcium from the ER to the cytosol. Conversely, calcium can passively leave the ER into the cytosol when IP3R channels open in dependence on calcium and the second messenger IP3 (1, 36, 37) associated with calcium-induced-calcium-release (CICR) (38). Furthermore other second messengers like cyclic ADP ribose (cADPR) (16) and nicotinic acid adenine dinucleotide phosphate (NAADP) (39) were found to influence calcium dynamics. Their effect is mediated by activation of the ryanodine receptor (RyR) which leads to calcium-release from intracellular stores (17, 40–43). In order to avoid an over-parametrization of whole cell calcium curves, which the present model focusses on, we restrict ourselves to the dynamics of IP3. The inclusion of cADPR and NAADP and their effect on RyR requires a more detailed data basis and should be addressed with a more complex model in the future.

#### 2.1.1 Calcium in the cytosol

The four sources and sinks of free cytosolic calcium *C* are described as

(1) $$\begin{array}{llll} \frac{dC}{dt} & =\frac{-1}{z_{\text{Ca}}F\left(1+B_{\text{C}}\right)}\left(\xi \rho_{\text{PMCA}}I_{\text{PMCA}}+\xi \rho_{\text{CRAC}}I_{\text{CRAC}}\right.\; & & \;\\ & \;\left.\;+\;\xi_{\text{ERC}}\rho_{\text{SERCA}}I_{\text{SERCA}}+\xi_{\text{ERC}}\rho_{\text{IP3R}}I_{\text{IP3R}}\right), \end{array}$$

where *z*_Ca_ = 2 is the valence of calcium ions, *F* the Faraday constant, ξ and ξ_ERC_ geometrical surface to volume ratios for PM [equation (16)] and ER membrane [equation (17)], respectively. ρ_X_ is the surface density and *I*_X_ the single transmembrane protein current, which are defined in the subsequent sections. By convention, positive ions that enter the cytosol are represented by negative electrical currents. *B*_C_ represents the cytosolic calcium-buffer in the rapid buffer approximation

(2) $$B_{\text{C}}=\frac{b_{0}K_{\text{b}}}{{\left(C+K_{\text{b}}\right)}^{2}}\;,$$

where *b*_0_ is the total buffer concentration, and *K*_b_ the calcium-buffer dissociation constant. The fraction of free calcium in the cytosol then reads

(3) $$f_{\text{C}}={\left[1+\frac{b_{0}}{C+K_{\text{b}}}\right]}^{-1}\;.$$

The main buffer within the cytosol is calmodulin (CaM) with 4 calcium binding sites per CaM. There is a diversity of measured CaM concentrations depending on cell type and organ (44–46). A realistic average value is 25 μM of CaM, corresponding to *b*_0_ = 100 μM of calcium binding sites. The dissociation constant *K*_b_ was determined by the required fraction of free calcium of *f*_C_ ≈ 0.1% in non-excitable cells (47), leading to *K*_b_ = 0.1 μM.

#### 2.1.2 Calcium in the ER

The dynamics of the calcium concentration in the ER *C*_ER_ is described by an equation analogous to equation (1)

(4) $$\frac{dC_{\text{ER}}}{dt}=\frac{\xi_{\text{ER}}\left(\rho_{\text{SERCA}}I_{\text{SERCA}}+\rho_{\text{IP3R}}I_{\text{IP3R}}\right)}{z_{\text{Ca}}F\left(1+B_{\text{C},\text{ER}}\right)}\;,$$

with a different geometrical factor ξ_ER_ [equation (18)], and a different calcium-buffer *B*_C,ER_ defined by

(5) $$B_{\text{C},\text{ER}}=\frac{b_{\text{ER},0}K_{\text{ER},\text{b}}}{{\left(C_{\text{ER}}+K_{\text{ER},\text{b}}\right)}^{2}}\;.$$

The fraction of free calcium in the ER reads

(6) $$f_{\text{C},\text{ER}}={\left[1+\frac{b_{\text{ER},0}}{C_{\text{ER}}+K_{\text{ER},\text{b}}}\right]}^{-1}\;.$$

The resting ER calcium level is *C*_ER,0_ = 400 μM (2) which holds true for Jurkat TCs considered here (34).

In the ER calcium is buffered by calsequestrin, with three high and three low-affinity binding sites (48), and by calreticulin, with two distinct domains, one with high-affinity (*K* = 0.01 mM) but low capacity (0.6–1 mol Ca^2+^/mol protein), and one with low-affinity (*K* = 2 mM) but high capacity (18 mol Ca^2+^/mol protein) (49). As calreticulin binds more than 50% of the luminal ER calcium (50) only this buffer is considered here. In pancreatic acinar cells it was estimated that 20-times more calcium would be free in the ER compared to the cytosol (47), suggesting *f*_C,ER_ ≈ 0.02. This is achieved by using *b*_ER,0_ = 30 mM and *K*_ER,b_ = 0.1 mM, which corresponds to an intermediate dissociation constant of both calcium binding domains.

### 2.2 IP3 dynamics

IP3 (*P*) is generated in a TCR- and calcium-dependent way described by

(7) $$\frac{dP}{dt}=\beta_{\text{P}}H\left(C,C_{\text{P}},n_{\text{P}}\right)T\left(t\right)-\gamma_{\text{P}}P\;,$$

where β_P_ is the production and γ_P_ the degradation rate. The Hill-function is defined as

(8) $$H\left(X,K,n\right)\equiv \frac{X^{n}}{X^{n}+K^{n}}\;,$$

where *K* is the concentration of *X* at which the Hill-function reaches its half value, and *n* the Hill-coefficient which determines the steepness of the Hill-function.

The degradation rate in equation (7) is determined by steady state conditions for IP3 in equation (35). The production rate is the tonic production rate and is modulated by increased calcium with the Hill-function in equation (7), leading to a positive feedback loop between calcium and IP3. β_P_ is fitted as described in Section 2.10 and mainly influences the speed of the early calcium response after TC stimulation.

The production is further modified by the time-dependent input function *T*(*t*) representing the degree of TCR stimulation of the cell. *T* = 1 is assumed in the resting state.

The resting concentration of IP3 *P*_0_ is identified as critical parameter. It strongly determines the responsivity of the cell via activation of IP3R (see Section 2.6). It was fitted as described in Section 2.10.

### 2.3 Membrane and reversal potentials

The resting membrane potential is set to *V* = −60 mV (51–53). It is assumed that the membrane potential is not changed by the calcium currents (*V* = *V*_0_) and that the electrical current corresponding to calcium fluxes in or out of the cell is equilibrated by other ions.

Further it is assumed that *V*_ER,0_ = *V*_0_ = −60 *mV*, thus, the ER and the cytosol are electrically equilibrated (54). ER calcium efflux may lead to small fluctuations (55) which are neglected. Thus, *V*_ER_ = *V* is assumed at all times.

In this approximation, the reversal potentials depend on the chemical gradient only. The Nernst-equation is used to calculate the reversal potential during dynamical changes of calcium concentrations:

(9) $$\begin{array}{llll} \bar{V_{\text{C}}} & =\frac{\mathrm{RT}}{z_{\text{Ca}}F}\ln\left(\frac{C_{\text{ext}}}{C}\right)-\Delta V_{\text{C}}\; & & \;\\ \bar{V_{\text{C},\text{ER}}} & =\frac{\mathrm{RT}}{z_{\text{Ca}}F}\ln\left(\frac{C_{\text{ER}}}{C}\right)-\Delta V_{\text{C},\text{ER}}\;, \end{array}$$

where *R* = 8.315 J/(K mol) the Rydberg (molar) gas constant, *T* = 310 K, and *F* the Faraday constant.

**Figure 2:**
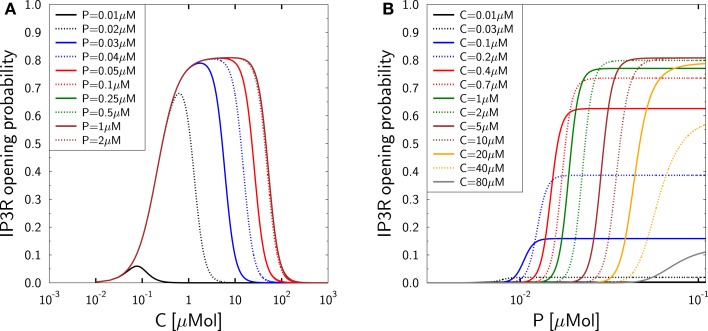
**Activation and inactivation of IP3R in dependence on calcium and IP3**. Reproduction of the experimental data (37) with the Mak–McBride–Foskett model equation (27). **(A)** IP3R opening probability *g*_IP3R_*h*_IP3R_ in dependence on free cytosolic calcium *C* for different IP3-concentrations *P*. **(B)** The same IP3R opening probability in dependence on IP3-concentration *P* for different free cytosolic calcium concentrations *C*.

For many channels, the real I-V-relationship is not linear as assumed for the currents *I*_X_ in equations (23) and (28). Therefore, the reversal potential is corrected for the CRAC-channel by a shift Δ*V*_C_ = 78 mV in order to achieve the correct linear extrapolation of the I-V-relation of CRAC-channels with a zero around $\bar{V_{\text{C}}}\approx 50\;\text{mv}$ [(34) Figure 1, (33) Figure 2]. This approximation is only valid for depolarization below *V* = 50 mV.

The reversal potential for ER calcium $\bar{V_{\text{C},\text{ER}}}$ is treated in complete analogy to the cytosolic case which leads to a correction of Δ*V*_C,ER_. As the value is not known it is derived using the fitting routine in Section 2.10.

### 2.4 TC geometry

Most measurements on TC calcium dynamics are performed in Jurkat TCs which are small but still larger than normal human blood derived TCs. In an approach based on ordinary differential equations the effect of an ion current onto the concentration of the ion in the cytosol or ER is not spatially resolved. While local calcium entry can induce transient high concentrations of calcium (22) the comparably small cytosolic volume of TCs justifies this approach for the description of whole cell calcium dynamics because local inhomogeneities will quickly equilibrate. In the model this is reflected as change of the average concentration. How an ion current changes the average calcium concentration depends on the geometry of the cell. In the dynamic equations for the ion concentrations the current *I*_X_ through an individual ion-conducting protein *X* is multiplied by the surface density ρ_X_. Thus the concentration change is derived from a current surface density. The latter has to be translated to the actual change in concentration by a surface to volume ratio, which is considered here.

Given a cell radius *R*_cell_, the cell volume *V*_cell_ and cell surface *A*_cell_ are known as well. However, the volume relevant to changes in concentration is not the cell volume *V*_cell_ but the cytosolic volume *V*_cyt_ which can be approximated as

(10) $$V_{\text{cyt}}=V_{\text{cell}}-{\tilde{V}}_{\text{ER}}-V_{\text{nucleus}}\;,$$

using the volumes of ER and nucleus. This is important because the nucleus, with a radius of

(11) $$R_{\text{nucleus}}=f_{\text{R}}R_{\text{cell}}$$

is substantially reducing the resulting cytosolic volume. *f*_R_ ≈ 0.8 is assumed for human TCs (56), and *f*_R_ ≈ 0.25 for Jurkat TCs. The volume of the ER is expressed as a fraction of the total cell volume

(12) $${\tilde{V}}_{\text{ER}}=f_{\text{V}}V_{\text{cell}}\;,$$

with *f*_V_ ≈ 0.1 (57). However, electron micrographs of TCs suggest that *f*_V_ ≈ 0.01 (25) which is used here. Taking this together, the cytosolic volume becomes

(13) $$V_{\text{cyt}}=V_{\text{cell}}\left(1-f_{\text{V}}-f_{\text{R}}^{3}\right)\;.$$

The surface of the ER is also needed in order to translate the current surface densities calculated on the ER surface into concentration changes in cytosol and ER. While the TC itself is approximated as a sphere, the ER is absolutely non-spherical. The exact surface of the ER is difficult to be measured and accordingly approximated as

(14) $$A_{\text{ER}}=f_{\text{A}}{Ã}_{\text{ER}}\;\equiv \;4\pi f_{A}{\left(\frac{3{\tilde{V}}_{\text{ER}}}{4\pi }\right)}^{2∕3}\;,$$

where ${Ã}_{\text{ER}}$ is the surface of a spherical ER with volume ${\tilde{V}}_{\text{ER}}$ [determined in equation (12)], and *f*_A_ is the fold increase of the ER surface with respect to the surface of a spherical ER. *f*_A_ = 30 was roughly estimated from the folding degree of the ER in electron micrographs of TCs (25). Note that only the product of *f*_A_ with $f_{\text{V}}^{2∕3}$ enters the model, such that both parameters are redundant and were only separated because of their physiological meaning.

The size of human blood TCs can be estimated starting from the capacity of *C*_m_ = 0.028 pF/μm^2^ (58, 59) and using the whole cell capacitance of *C*_cell_ = 2 pF [(60), p. 606]. *C*_cell_ = 1.7 pF was found in Fomina et al. (14). Using *C*_cell_/*A*_cell_ = *C*_m_ this yields a radius

(15) $$R_{\text{cell}}=\sqrt{\frac{C_{\text{cell}}}{4\pi C_{\text{m}}}}$$

and the resulting *R*_cell_ ≈ 2.4 μm corresponds to *A*_cell_ = 72.4 μm^2^. The experiments described below were performed with Jurkat TCs and the same authors determined the cell volume to *V*_cell_ = 2 pl (12). This determines the values *R*_cell_ = 8 μm and *A*_cell_ = 804.2 μm^2^ used in the present simulations.

Given the cell radius *R*_cell_, the fractions *f*_V_ and *f*_R_, as well as the factor *f*_A_, the surface to volume ratios required in equations (1) and (4) can be calculated by

(16) $$\begin{array}{ll} \xi & =\frac{A_{\text{cell}}}{V_{\text{cyt}}} \end{array}$$

(17) $$\begin{array}{ll} \xi_{\text{ERC}} & =\frac{A_{\text{ER}}}{V_{\text{cyt}}} \end{array}$$

(18) $$\begin{array}{ll} \xi_{\text{ER}} & =\frac{A_{\text{ER}}}{{\tilde{V}}_{\text{ER}}} \end{array}$$

with

(19) $$\begin{array}{ll} A_{\text{cell}} & =4\pi R_{\text{cell}}^{2} \end{array}$$

(20) $$\begin{array}{ll} V_{\text{cyt}} & =\frac{4}{3}\pi R_{\text{cell}}^{3}\left(1-f_{\text{V}}-f_{\text{R}}^{3}\right) \end{array}$$

(21) $$\begin{array}{ll} {\tilde{V}}_{\text{ER}} & =\frac{4}{3}\pi f_{\text{V}}R_{\text{cell}}^{3} \end{array}$$

(22) $$\begin{array}{ll} A_{\text{ER}} & =4\pi f_{\text{A}}{\left(\frac{3{\tilde{V}}_{\text{ER}}}{4\pi }\right)}^{2∕3}\;. \end{array}$$

### 2.5 CRAC-channel

The open CRAC-channel current is determined by the electrochemical gradient

(23) $$I_{\text{CRAC}}=\bar{g_{\text{CRAC}}}\left(V-\bar{V_{\text{C}}}\right)\;.$$

This approach closely follows the model of Martin et al. (22). The validity of the Ohm’s law approximation is only guaranteed within limited ranges of membrane potentials.

#### 2.5.1 Single channel conductance

The single channel CRAC conductance was found to be extremely small in the order of $\bar{g_{\text{CRAC}}}=2\;\text{fs}$ (61).

#### 2.5.2 CRAC recruitment

The density of active CRAC-channels, estimated by the steady state CRAC-channel current, is a dynamic function of the ER-calcium concentration [(34) Figure 1C] described by

(24) $$\frac{d\rho_{\text{CRAC}}}{dt}=\frac{\bar{\rho_{\text{CRAC}}}-\rho_{\text{CRAC}}}{\tau_{\text{CRAC}}}\;,$$

where

(25) $$\begin{array}{llll} \bar{\rho_{\text{CRAC}}} & =\rho_{\text{CRAC}}^{-}+\left(\rho_{\text{CRAC}}^{+}-\rho_{\text{CRAC}}^{-}\right)\; & & \;\\ & \;\left(1-H\left(C_{\text{ER}},C_{\text{CRAC}},n_{\text{CRAC}}\right)\right), \end{array}$$

with *C*_CRAC_ = 169 μM and *n*_CRAC_ = 4.2. To our knowledge, this is the first time that the surface density of active CRAC-channels in the PM is modeled as a dynamic quantity.

When estimating the same quantity from the degree of STIM1-redistribution, a rather similar relation is found with *C*_CRAC_ = 187 μM and *n*_CRAC_ = 3.8 [(34), Figure 2]. The uniformity of both curves supports the view that CRAC-channels are recruited and open in response to ER calcium depletion (34). It can be deduced from the similarity of both curves that the opening dynamics is substantially faster than the redistribution of STIM1. As no opening dynamics of the CRAC-channel is included in the model, the dynamics of the current itself and not of STIM1-redistribution is used.

#### 2.5.3 CRAC density

In equation (25) $\rho_{\text{CRAC}}^{\pm }$ are the upper and lower limits of possible active CRAC densities. The resting value ρ_CRAC,0_ is not known from experiment and is determined by parameter fitting to calcium dynamics upon TCR stimulation (Section 2.10).

The density of CRAC-channels upon activation with PHA increased about 9-fold (33) which constraints $\rho_{\text{CRAC}}^{+}$. A 10-fold increase has been reported for the whole cell CRAC current in response to stepwise reduced *C*_ER_ [Figure 1C in Luik et al. (34)]. These findings translate into the condition

(26) $$\rho_{\text{CRAC}}^{+}=f_{\text{CRAC}}\rho_{\text{CRAC},\text{0}}\;.$$

where $\rho_{\text{CRAC}}^{+}$ was determined by parameter fitting within the experimental boundaries in Section 2.10. A value for $\rho_{\text{CRAC}}^{-}$ is not known and is determined by the steady state condition equation (36).

#### 2.5.4 CRAC time scales

The time scale of CRAC recruitment can be estimated from the rising time of calcium curves which provides an upper bound of τ_CRAC_ < 100 s for the activation time. It is likely that this time is associated with CRAC recruitment rather than with CRAC opening because opening time scales are typically much shorter. The time scale of CRAC recruitment is set to τ_CRAC_ = 5 s. Larger values could also be used as the fit was insensitive to τ_CRAC_. Inactivation of CRAC-channels is difficult to be assessed (62). As the time scale of inactivation is in the order of 1000 s (14) and thus longer than the typical experimental durations used here, the present model ignores CRAC inactivation and assumes that the reduction of active CRAC-channels is a secondary effect of *C*_ER_ recovery.

### 2.6 IP3R in the ER

The ER releases its calcium content if activated by IP3 and cytosolic calcium (63, 64). The release of calcium from intracellular stores is based on the opening dynamics of RyR and IP3R in the membrane of the ER. TCs express both, RyR and IP3R and even different subtypes of them.

IP3Rs have binding sites for IP3 and calcium and exhibit complex forms of cooperativity (65). For the present purpose the heuristic description of IP3R activation and inhibition is sufficient. The characteristic feature of the IP3R conductance is a calcium-dependent log-bell-shaped opening probability curve (63) which has been measured for ER vesicles from canine cerebellum and further reviewed in Foskett et al. (38). The open probability curve was best fitted by the product of an activating and an inhibiting Hill-function, both with Hill-coefficient 2 (63).

#### 2.6.1 Open probability

TCR signaling leads to the generation of IP3, the ligand of the IP3R, which modulates the open probability of IP3R and is described as the product of an activation term *g*_IP3R_ and an inactivation term *h*_IP3R_. The properties of single channel openings were quantitatively determined in *Xenopus laevis* oocytes (37, 38) and we assume that the single channel properties are transferable to TCs. The measured dynamics are well described by the previously published Mak–McBride–Foskett model (37, 38).

(27) $$\begin{array}{llll} g_{\text{IP3R}} & =g_{\text{IP3R},\text{max}}H\left(C,C_{\text{IP3R},\text{act}},n_{\text{IP3R},\text{act}}\right)\; & & \;\\ h_{\text{IP3R}} & =H\left(C_{\text{IP3R},\text{inh}},C,n_{\text{IP3R},\text{inh}}\right)\; & & \;\\ C_{\text{IP3R},\text{inh}} & =\bar{C_{\text{IP3R},\text{inh}}}H\left(P,P_{\text{IP3R},\text{C}},n_{\text{IP3R},\text{C}}\right). \end{array}$$

The according parameters were obtained from a data fit (37) to be *g*_IP3R,max_ = 0.81, *C*_IP3R,act_ = 0.21 μM, *n*_IP3R,act_ = 1.9, *n*_IP3R,inh_ = 3.9, $\bar{C_{\text{IP3R,inh}}}$ = 52 μM, *n*_IP3R,C_ = 4, and the IP3-concentration of half-activation *P*_IP3R,C_ = 0.050 μM.

The dependencies of the model equation (27) on calcium and IP3 are depicted in Figure 2 and correctly reproduce the measurements in Mak et al. (37) suggesting that the modulating effect of IP3 is mediated by IP3R inactivation (38). At low calcium this effect is hardly visible and IP3R activation remains unaffected by changes in IP3 for resting concentrations beyond 50 nM. The dynamic IP3 range is between 100 nM and 1 μM (see (66), Table 1), a regime of IP3 at which the IP3R-type1 exhibits saturation (27, 67). We do not aim at resolving whether the resting IP3 is lower in TCs or whether the IP3R characteristics are different in TCs, such that the DeYoung–Keizer model should be employed instead (27, 65). It is assumed that the resting concentration of IP3 is in the range of 5–10 nM which ensures that an increased IP3-concentration has an impact on the IP3R opening probability as depicted in Figure 2.

#### 2.6.2 IP3R calcium current

The steady state activation function (Figure 2) can be used to define the calcium current through the IP3R which follows the electrochemical gradient between the cytosol and the ER

(28) $$I_{\text{IP3R}}=\bar{g_{\text{IP3R}}}g_{\text{IP3R}}h_{\text{IP3R}}\left(V-V_{\text{ER}}-\bar{V_{\text{C},\text{ER}}}\right),\;$$

with $\bar{g_{\text{IP3R}}}=0.064\mathrm{pS}$. Note that the conductance differs between tissues (38). *V* − *V*_ER_ is the potential difference between ER and cytosol, and $\bar{V_{\text{C},\text{ER}}}$ is the ER-reversal potential for calcium, calculated from the Nernst-equation equation (9). We assume an electrical equilibrated relation of cytosol and ER such that *V* = *V*_ER_ holds true.

#### 2.6.3 (In)activation dynamics

The activation and inactivation factors *g* and *h* are treated dynamically and approach equation (27) in steady state, while the adaptation of *C*_IP3R,inh_ in equation (27) is treated in quasi steady state:

(29) $$\begin{array}{llll} \frac{dg_{\text{IP3R}}}{dt} & =\frac{g_{\text{IP3R},\text{max}}H\left(C,C_{\text{IP3R},\text{act}},n_{\text{IP3R},\text{act}}\right)-g_{\text{IP3R}}}{\tau_{\text{IP3R}}}\; & & \;\\ \frac{dh_{\text{IP3R}}}{dt} & =\frac{H\left(C_{\text{IP3R},\text{inh}},C,n_{\text{IP3R},\text{inh}}\right)-h_{\text{IP3R}}}{\theta_{\text{IP3R}}}\;. \end{array}$$

#### 2.6.4 Activation time

Activation time scales can be determined from Mak and Foskett (68), Figure 5, and are in the range of less than 5 and 20 ms for depolarizations to 20 and 40 mV. Two exponentials were needed to fit the opening frequency. Using rat hepatocytes the activation and inactivation time scales were found to depend on the IP3-levels (69): activation varied between 100 and 500 ms for 10 μM and 400 nM of IP3, respectively (see Figure 1 therein). The time delay reported in Marchant and Taylor (69) is consistent with the IP3-dependent time delay of channel opening of 1 s > τ_IP3R_ > 100 ms using basophilic leukemia cells from rats (70). As the model focusses on calcium dynamics on the scale of minutes, a constant τ_IP3R_ = 100 ms is assumed.

#### 2.6.5 Inactivation time

Onset of inactivation happens in less than 2 min (68). A slow and a fast current were distinguished (69). The fast current inactivates on a time scale of 200–450 ms, the slow one between 1 and 6 s (see Figure 2 in the same publication). The authors attribute the fast time scale to inactivation of IP3R and the slow one to the depletion of the calcium content in the ER. Accordingly, only the fast time scales are relevant for the single IP3R, and θ_IP3R_ = 300 ms is assumed.

#### 2.6.6 Calmodulin dependence

It was found that the calcium-release from ER is reduced for high concentrations of the calcium-buffer calmodulin (71). Such a dependence is neglected in the present model.

#### 2.6.7 IP3R density

The IP3R density on Jurkat TCs is not known and is determined using steady state condition equation (34).

### 2.7 Plasma-membrane calcium-ATPase

Plasma-membrane calcium-ATPase is an ATP-driven calcium pump which extrudes calcium from the cell to the ES. It was characterized in TCs (12). In a first attempt the dependence on the ATP concentration is ignored and assumed to be large enough in order to make the pump work optimally. In this case the activity is mainly dependent on the calcium concentration in the cell. A suitable modeling approach is

(30) $$I_{\text{PMCA}}=\bar{I_{\text{PMCA}}}g_{\text{PMCA}}$$

with

(31) $$\frac{dg_{\text{PMCA}}}{dt}=\frac{H\left(C,C_{\text{PMCA}},n_{\text{PMCA}}\right)-g_{\text{PMCA}}}{\tau_{\text{PMCA}}}.\;$$

The current $\bar{I_{\text{PMCA}}}$ is positive as it carries calcium out of the cell. The Hill-coefficient was determined to be *n*_PMCA_ = 2 (72).

#### 2.7.1 Turn-over rate

The turn-over rate of PMCA is approximately 30 Hz (73) which corresponds to an activity rate of *k*_a_ = 0.03/ms which is also used in Sherman et al. (74). This turn-over rate can be translated into an electrical current by using that every pumping event corresponds to the flow of 2 electrical charges which yields $\bar{I_{\text{PMCA}}}=z_{\text{Ca}}{\mathrm{ek}}_{\text{a}}=60\cdot 1.6\cdot 10^{-19}$ C/s ≈ 10^−17^ A = 10^−5^ pA.

#### 2.7.2 Calcium-dependent activation

**Figure 3:**
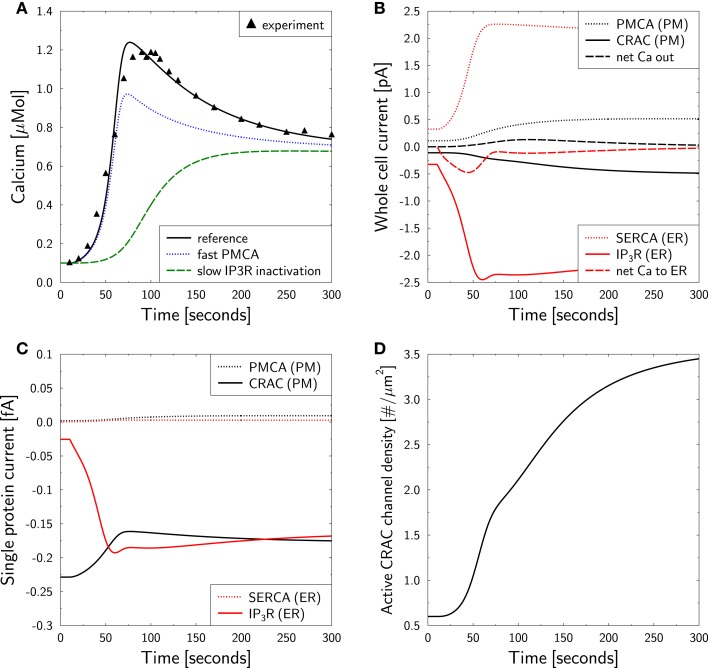
**Calcium dynamics in response to TCR stimulation**. Simulation of the experiment in Figure 1A in Bautista et al. (12). **(A)** The cell is in steady state until *t* = 10 s when stimulation is started by setting *T*(*t*) = 1.6 in equation (7). Stimulation is kept constant throughout the simulation. *Reference* is the simulation used as a basis for all other simulations in the paper and is compared to the experimental result [black triangles read off Figure 1A in Bautista et al. (12)]. For *fast PMCA* a value of 1 ms was used for τ_PMCA_. For *slow IP3R inactivation* a value of 300 s was used for θ_IP3R_. **(B)** Whole cell currents show an initial release of calcium from the ER which is followed by a CRAC inward current. Negative currents are calcium currents into the cytosol. **(C)** Single transmembrane protein currents. **(D)** Dynamic response of active CRAC-channel density.

Typical values for the half-activation calcium concentration are *C*_PMCA_ = 0.1 μM (at 540 nM calmodulin, see e.g., (75), Figure 3). The isoforms 2a and 2b exhibit *C*_PMCA2ab_ < 0.1 μM. The predominant isoform of PMCA in Jurkat TCs is 4b [(76), Figure 6B]. *C*_PMCA4b_ ≈ 0.2 μM was found in Jurkat TCs [Figure 2 in Caride et al. (76)] and is used here.

#### 2.7.3 Calmodulin-dependent activation

Note that the values of half-activation also depend on the calmodulin concentration (75– 77). For calmodulin concentrations above 1 μM (which is exclusively the case in all present simulations) full activation of all isoforms is ensured (75, 78). Hence, the dependence on calmodulin is weak in this regime and is neglected.

#### 2.7.4 Delay of activation

Binding of calcium to PMCA is a comparably fast process with a rate constant >3 per second (79). However, the activity of PMCA is delayed in some isoforms including the isoform 4b (76) which is relevant for TCs. The rate constant of PMCA activation upon stimulation with 500 nM of free calcium was in the range of 0.02 per second (76), which leads to a delay of PMCA activation in the range of τ_PMCA_ = 50 s in equation (31). This delay was associated with a calmodulin and calcium-dependent activation (76). However, as the exact mechanism is not known this delay is modeled in equation (31) on a phenomenological basis.

#### 2.7.5 PMCA density

For TCs no precise value of the protein density is known and the value is determined by the steady state condition equation (33).

### 2.8 Sarco/endoplasmic reticulum calcium-ATPase

The calcium level in ER is kept high with the help of SERCA calcium pumps. The activity of SERCA is assumed to rapidly adapt to the present calcium concentration in the cytosol and can be described by a Hill-function.

(32) $$I_{\text{SERCA}}=\bar{I_{\text{SERCA}}}H\left(C,C_{\text{SERCA}},n_{\text{SERCA}}\right).\;$$

In every turn-over cycle two calcium ions are transported per ATP (80). There are different subtypes of SERCA whose properties differ. In Jurkat TCs as well as in human tonsil lymphocytes the dominant isoform is SERCA2b (81).

#### 2.8.1 Turn-over rate

The turn-over rates of most isoforms are in the range of *k* = 10 Hz (i.e., $\bar{I_{\text{SERCA}}}$ = α_SERCA_*z*_Ca_*ek* = 6 ⋅ 10^−6^ pA). For SERCA2b *k*_2b_ = 5 Hz was reported (82) which implies the value $\bar{I_{\text{SERCA}}}=3\cdot 10^{-6}$ pA used in the model.

#### 2.8.2 Calcium-dependent activation

**Figure 4:**
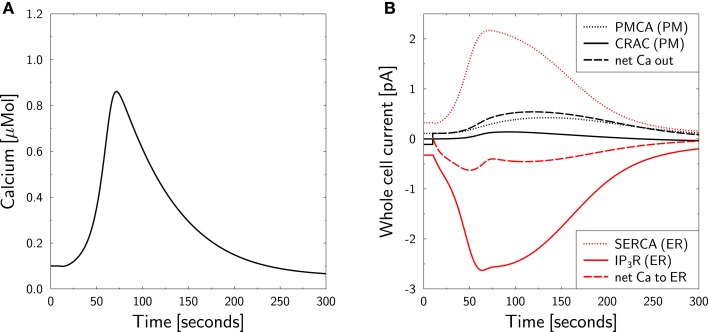
**Calcium dynamics in response to TCR stimulation at zero external calcium**. Simulation of the experiment in Figure 2A in Bautista et al. (12). **(A)** The cell is in steady state until *t* = 10 s, when stimulation is started by setting *T*(*t*) = 1.6 in equation (7) and external calcium is set to equation (38). Stimulation and external calcium are kept constant throughout the simulation. **(B)** Whole cell currents show the almost complete inhibition of CRAC currents. The calcium peak (left) is generated by ER calcium only. Negative currents are calcium currents into the cytosol.

For the SERCA isoforms 1, 2a, and 2b the half-activation *C*_SERCA_ = 0.4 μM and the Hill-coefficient *n*_SERCA_ = 2 are a good approximation [(82) Figure 4]. The half-activation of SERCA3 is around 1μM with the same Hill-coefficient (82). SERCA2b is an isoform active at relatively low calcium concentrations (82). Specifically, in Jurkat TCs as well as in human tonsil lymphocytes, the dominant isoform SERCA2b was characterized with *n*_SERCA2b_ = 2.0 and *C*_SERCA2b_ = 0.25 μM (81), which is used here.

#### 2.8.3 SERCA density

Even though the expression of SERCA was shown to be modulated upon activation (83), the expression density of SERCA within ER is not known and is determined by parameter fitting in Section 2.10.

### 2.9 Steady state determines protein densities

The resting state of the TC is determined by setting the dynamics in equations 1, 4, 7, and 24 to zero. Accordingly, the equations for *C*, *C*_ER_, *P*, and ρ_CRAC_, read:

(33) $$\begin{array}{ll} \rho_{\text{PMCA}} & =-\frac{\rho_{\text{CRAC},0}I_{\text{CRAC},0}}{I_{\text{PMCA},0}} \end{array}$$

(34) $$\begin{array}{ll} \rho_{\text{IP3R}} & =-\frac{\rho_{\text{SERCA}}I_{\text{SERCA},0}}{I_{\text{IP3R},0}}\; \end{array}$$

(35) $$\begin{array}{ll} \gamma_{\text{P}} & =\frac{\beta_{\text{P}}H\left(C_{0},C_{P},n_{P}\right)}{P_{0}} \end{array}$$

(36) $$\begin{array}{llll} \rho_{\text{CRAC}}^{-} & =\frac{\rho_{\text{CRAC},0}}{H\left(C_{\text{ER},0},C_{\text{CRAC}},n_{\text{CRAC}}\right)}\; & & \;\\ & \;\left[1-f_{\text{CRAC}}\left(1-H\left(C_{\text{ER},0},C_{\text{CRAC}},n_{\text{CRAC}}\right)\right)\right], \end{array}$$

where *I*_X,0_ denotes the currents with all quantities *X* in the resting configuration *X*_0_. The parameters of the model are summarized in Table 1.

**Table 1:** **List of parameters, values, and references**.

Parameter	Meaning	Value	ΔQI (%)	Comments and reference
**CELL GEOMETRY**				
*R*_cell_	TC radius	8 μm	4.0	Fixed from Jurkat TC (12 )
*f*_R_	Nucleus radius fraction of *R*_cell_	0.25	0.1	Fixed, Section 2.4
*f*_V_	ER volume fraction	0.01	2.8	Fixed (25 )
*f*_A_	ER surface, fold of spherical	30	3.5	Fixed, Section 2.4
*C*_m_	Membrane capacitance	28 fF/μm^2^	0.0	Fixed (58, 59)
**IONS AND POTENTIAL**				
*T*	Temperature	310 K	–	Fixed, used in equation (9)
*V*_0_	Resting membrane potential	−60 mV	–	Fixed (51, 52, 53 )
*V*_ER,0_	Resting ER potential	−60 mV	–	Fixed = *V*_0_ (54)
*C*_0_	Resting calcium	0.1 μM	–	Fixed (12 )
*C*_ER,0_	Resting ER calcium	0.4 mM	4.0	Fixed in range 0.1–0.8 mM (2 )
*C*_ext_	Extracellular calcium	2 mM	0.0	Fixed by experimental settings
Δ*V*_C_	Reversal potential shift	78 mV	0.3	Fixed (33, 34 )
Δ*V*_C,ER_	ER-reversal potential shift	63 mV	176	Variable
**CALCIUM-BUFFER**				
*b*_0_	Cytosolic calcium-buffer	100 μM	3.6	Fixed (44, 45, 46 )
*K*_b_	Buffer dissociation constant	0.1 μM	0.2	Fixed by *f*_C_ = 0.1% in equation (3)
*b*_ER,0_	ER calcium-buffer	30 mM	4.3	Fixed by *f*_C,ER_ = 20*f*_C_ (47)
*K*_ER,b_	ER buffer dissociation constant	0.1 mM	1.2	Fixed (49 )
**SECOND MESSENGERS**				
*P*_0_	Resting IP3	8.7 nM	318	Variable
β_P_	IP3 production rate	0.6 nM/s	6.5	Variable
γ_P_	IP3 degradation rate	0.01149/s	–	Fixed by steady state equation (35)
*C*_P_	Calcium of half IP3 production	0.5 μM	19.4	Fixed (85 )
*n*_P_	IP3 production Hill-coefficient	1	21.2	Fixed
**TRANSMEMBRANE PROTEIN DENSITIES**				
ρ_IP3R_	ER-IP3R density	11.35/μm^2^	–	Fixed by steady state equation (34)
ρ_SERCA_	ER-SERCA density	700/μm^2^	3.7	Variable
ρ_PMCA_	PMCA density	68.57/μm^2^	–	Fixed by steady state equation (33)
ρ_CRAC,0_	Resting active CRAC density	0.6/μm^2^	1.0	Variable
$\rho_{\text{CRAC}}^{+}$	Max active CRAC density	3.9/μm^2^	6.5	Variable, range from Luik et al. (34)
$\rho_{\text{CRAC}}^{-}$	Min active CRAC density	0.5115/μm^2^	–	Fixed by steady state equation (36)

Fixed parameters were determined either by direct measurement, by indirect constraints, or using steady state conditions. Variable parameters were subject to the fitting algorithm described in Section 2.10. ΔQI measures the sensitivity of QI in equation (37) for changed parameter values: each parameter is increased by 10% and the percentage of the change in QI is provided. Values below 0.05% are given as 0.0%.

### 2.10 Numerical solution and parameter fitting

The model defined by the equations (1, 2, 4, 5, 7, 8, 16–25, 27–32) was implemented as C^++^-code and solved using a self-written 4th-order Runge–Kutta algorithm with adaptive stepsize control.

As not all parameters could be determined by steady state conditions or by experimental constraints, Figure 1A in Bautista et al. (12) was used to determine the remaining free parameters. We used a two-step fitting procedure: at first, the differential evolution algorithm defined in Storn and Price (84) was incorporated into the C^++^-code of the model on the basis of all parameters in Table 1 that were not determined by steady state conditions. The parameters were varied within hard-coded boundaries dictated by experimental constraints (when available). The quality of the fit to the calcium data in Bautista et al. (12) was measured as the mean square deviation

(37) $$QI=\frac{1}{N}\sqrt{\sum_{i=1}^{N}\frac{{\left(X_{i}-E_{i}\right)}^{2}}{E_{i}^{2}}},$$

with *X*_i_ and *E*_i_ representing the simulation and experimental values, respectively. In a second step, the first approximative fit was subject to a sensitivity analysis in which each parameter was varied by 10% while monitoring the effect on QI. The three unknown protein densities ρ_SERCA_, ρ_CRAC,0_, and $\rho_{\text{CRAC}}^{+}$, two sensitive IP3 related parameters *P*_0_ and β_P_, as well as the very sensitive parameter Δ*V*_C,ER_ were used for fine-tuning the initial parameter fit with the same differential evolution algorithm. These fit parameters are marked *variable* in Table 1. The final fit reached QI = 0.100197 with *N* = 22. The sensitivity analysis was repeated for the final fit and the impact of each parameter on QI in equation (37) is provided in Table 1.

All subsequently described simulations are started with the cell in steady state as defined by the hard-coded equations (33–36). Starting from these initial conditions, the respective stimulation protocols are applied as described in the results section.

## 3 Results

In the methods section single protein characteristics were summarized and specific mathematical models capturing their main properties were proposed or cited. The models for the single proteins were combined to a whole cell model and the unknown parameters were determined using steady state conditions or by data fitting as described in Section 2.10. In this section, we replicate specific experimental setups described in the literature *in silico* and analyze the calcium dynamics from the perspective of the model.

### 3.1 TCR stimulation

The introduced TC-model is used to investigate the experiments of Bautista et al. (12) in Jurkat TCs. TC activation by stimulation of TCR induces an intracellular rise of second messengers like cADPR, NAADP, and IP3. In the present model this rise is collectively reflected in equation (7) for IP3. The IP3 signal activates IP3R and by this induces a calcium-release from the ER. The positive feedback loop of CICR leads to even more calcium-release from the ER, which in turn reduces the ER calcium concentration *C*_ER_. CRAC is activated in a *C*_ER_-dependent manner, as represented by equation (24). The rising cytosolic calcium is cleared by PMCA and the ER is refilled by SERCA, both being ATP-dependent processes.

Using 2 mM of external calcium a cell in resting state was activated with OKT3 via TCR [see Figure 1A in Bautista et al. (12)]. This induced a calcium peak to more than 1 μM within 50–100 s, which subsequently relaxed to a plateau level of ≈0.7 μM over the following 200 s. This behavior is well reproduced by the model (Figure 3A) and was used to determine the unknown parameters (Table 1). The peak height relies on both, on a proper activation of IP3R and CRAC. The choice of *P*_0_ turned out to be rather important in order to guarantee a proper activation of IP3R. The maximum activated CRAC density was essential for the height of the plateau. A value of *f*_CRAC_ = 6.5 was found to correctly reproduce the plateau height as measured in Bautista et al. (12).

Bautista et al. reported that the delay of PMCA activation is responsible for the calcium overshoot (12). Accordingly, we investigated whether this conclusion is supported by the model. For that purpose we reduced τ_PMCA_ from 50 s (76) to 1 millisecond in the otherwise unaltered simulation. This modification does not touch the steady state configuration, such that all other parameters remained unchanged. The model still generated an overshoot of calcium, however, with a reduced amplitude (Figure 3A, blue dotted line). The height of the plateau as well as the relaxation time from peak to plateau remained unchanged. Thus, the simulation supports an influence of the PMCA delay onto the overshoot, but it turned out to not be essential for its existence.

This surprising result led to the question whether a different delay in the model could explain the calcium overshoot. All delays were tested and the only delay with impact on the overshoot was IP3R inactivation, i.e., the parameter θ_IP3R_ (Figure 3A, green dashed line). In conclusion, the model suggests that intrinsic properties of the IP3R and not the delay of PMCA activation are responsible for the calcium overshoot upon TCR stimulation.

Next, the model is used for an analysis of the currents which are associated with the different phases of the calcium dynamics (Figure 3B). The IP3-current (red full line) is clearly the largest and also persists beyond the overshoot of calcium due to SERCA activity (red dotted line). The calcium peak induces a PMCA current (black dotted line) that drives the calcium out of the cell (black dashed line). Thus, the CRAC current (black full line), induced by the loss of calcium in the ER, does not even induce a net flow of calcium into the cell (black dashed line) but just prevents the cell from running out of calcium. This model result suggests that the role of CRAC is the stabilization of the cell rather than its activation, which is mostly mediated by calcium from the ER.

The single protein currents (Figure 3C) show that in the model IP3R currents react much more dynamic than CRAC currents. The increased cytosolic calcium even reduces the CRAC currents on the single channel level. However, the reduced ER calcium level induces a strong increase in the active CRAC-channel density (Figure 3D). Thus, the increased whole cell CRAC current seen in Figure 3B is not a result of single channel responses but of a changed channel density.

### 3.2 TCR stimulation with zero external calcium

Zero external calcium experiments aim at suppressing CRAC currents in order to investigate ER calcium currents in response to TCR stimulation. TCR stimulation of Jurkat TCs hold at zero external calcium results in an intracellular calcium peak after 50–100 s with reduced amplitude in comparison to stimulation with normal external calcium [Figure 2A in Bautista et al. (12)]. The increased calcium is cleared below baseline level within 100–200 s.

In the model, the Nernst-equation prohibits the use of zero external calcium conditions. At very low calcium concentrations the Nernst-equation loses its validity and the reversal potential diverges. Therefore, external calcium is set to the concentration $C_{ext}^{\;*}$ at which the CRAC current vanishes in resting state:

(38) $$C_{\text{ext}}^{*}=C_{0}\exp\left\{\frac{\left(V_{0}-\Delta V_{\text{C}}\right)z_{\text{Ca}}F}{\mathrm{RT}}\right\}\;.$$

This mimicks the suppression of CRAC currents as intended in the experiment. The *in silico* result is shown in Figure 4. The CRAC current vanishes at resting state (Figure 4B, black full line). This is approximately true during the whole experiment, such that the method for mimicking the zero external calcium experiment appears appropriate.

The calcium peak (Figure 4A) is lower than the one found in Figure 3 which is qualitatively consistent with the experimental result (12). Furthermore, the time scales of calcium rise and clearance are perfectly matched between simulation and experiment. Even the overshoot of clearance below the baseline calcium level is fully reproduced. However, quantitatively, the peak is higher in theory than in experiment. As the *in silico* peak is exclusively generated by the ER, one might hypothesize that the ER is too big or its calcium content is too high. As these parameters were already chosen comparably low (Table 1), it is more likely that the lack of external calcium influences the stimulation of the cell. The peak size can be reduced to the measured amplitude by reducing the stimulation *T* from 1.6 to 1.25 (not shown).

### 3.3 Block of SERCA at zero external calcium

Thapsigargin (TG) is frequently used to block SERCA activity as it prevents calcium uptake by the ER and leads to a continuous reduction of ER calcium. As low ER calcium recruits and activates CRAC-channels, this would lead to a strong influx of extracellular calcium. In order to prevent this according ER depletion and CRAC activation experiments are performed in zero calcium medium. This strategy was used in Jurkat TCs to generate a cell state in which the ER is mostly void of calcium and CRAC-channels are recruited and activated to a maximum (12, 13, 25, 86). It was reported that this procedure leads to a transient calcium peak of about 0.5 μM after more than 100 s which is slowly cleared and reaches calcium levels below the resting level [Figure 1A in Quintana et al. (86)].

**Figure 5:**
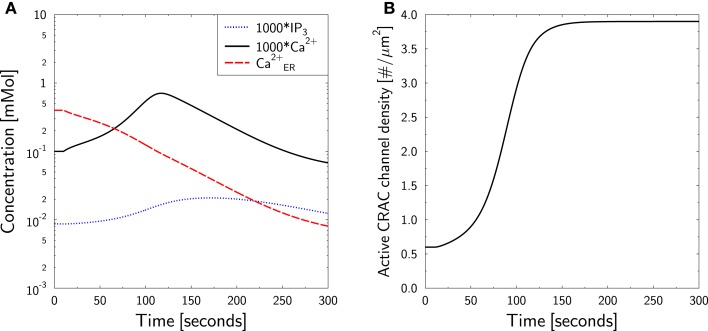
**Calcium dynamics in response to TG SERCA block at zero external calcium**. Simulation of the experiment in Figure 1A in Bautista et al. (86). **(A)** The cell is in steady state until *t* = 10 s, when SERCA block is applied and external calcium is set to equation (38). Block and external calcium are kept constant throughout the simulation. *P* (blue dotted line), *C* (black full line), and *C*_ER_ (red dashed line) are shown. Note the factor 1000 applied to *P* and *C*. **(B)** The time course of active CRAC density ρ_CRAC_.

Having established a strategy [equation (38)] for mimicking a medium with zero calcium, a SERCA block is performed *in silico* by setting *I*_SERCA_ = 0 at *t* = 10 s. No TCR stimulation was applied. The measured dynamics are well reproduced without any further parameter fitting (Figure 5A, black full line). Furthermore, the intended depletion of ER is achieved (Figure 5A, red dashed line): a continuous reduction of ER calcium is observed. Note that also IP3 exhibits some dynamics (Figure 5A, blue dotted line) which further accelerates ER calcium loss by activation of IP3R. As expected, the reduced ER calcium leads to the recruitment of active CRAC-channels (Figure 5B).

### 3.4 Block of PMCA in a TG treated TC

As the TG-mediated SERCA block works *in silico* (Figure 5), the role of PMCA in the clearance of cytosolic calcium is investigated. Following Figure 6 in Bautista et al. (12) TG was applied in a zero calcium medium as in Figure 5. Then a pulse of 2 mM external calcium was applied for 50 s. This induces a steep rise in calcium which is also steeply cleared again. Together with Lanthan (La^3+^), a PMCA, and CRAC inhibitor (2), the clearance of such a calcium peak was substantially slower (12). A block of PMCA alone by carboxyeosin led to an only weakly modified clearance time, without a return to baseline levels within 300 s.

**Figure 6:**
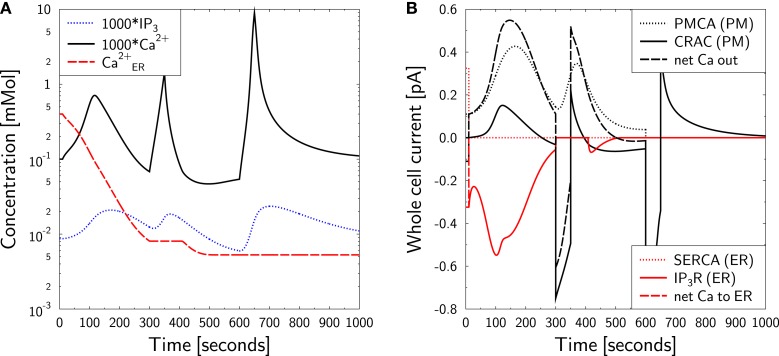
**Calcium dynamics in response to SERCA and PMCA block at zero external calcium**. Simulation of the experiment in Figures 6C,D of Bautista et al. (12). **(A)** The cell is in steady state until *t* = 10 s, when SERCA block is applied and external calcium is set to equation (38). At *t* = 300 s 2 mM external calcium are applied for 50 s followed by a switch back to equation (38). The procedure is repeated at *t* = 600 s. In addition, *I*_PMCA_ is set to zero for the remaining simulation time. SERCA block is kept throughout the simulation. *P* (blue dotted line), *C* (black full line), and *C*_ER_ (red dashed line) are shown. Note the factor 1000 applied to *P* and *C*. **(B)** The CRAC density was increased during the first 300 s by ER calcium depletion with TG at zero external calcium [**(A)** red dashed line]. The whole cell currents show a sudden CRAC current (black full line) upon restoration of external calcium to 2 mM, which is cleared by PMCA activity (black dotted line) after return to zero external calcium. When PMCA is blocked in addition (at *t* = 600 s), the calcium clearance is slower (black full line). Negative currents are calcium currents into the cytosol.

The same protocol is applied in the model (Figure 6). As before, ER calcium is depleted (Figure 6A, red dashed line) and the active CRAC density is increased correspondingly (as in Figure 5B). The initial free cytosolic calcium peak (Figure 6A, black full line) is the same as in Figure 5A. Upon increasing external calcium to 2 mM for 50 s at *t* = 300 s, a strong CRAC current is induced (Figure 6B, black full line), which steeply increases cytosolic calcium (Figure 6A, black full line). This calcium is also quickly cleared upon return to the mimicked zero calcium medium. Calcium clearance is dominated by the PMCA current (Figure 6B, black dotted line). However, in the model a small CRAC current is observed supporting extrusion of calcium out of the cell in the case of zero external calcium concentration. Upon permanent block of PMCA and repetition of the transient stimulation by external calcium, it is this backward CRAC current that clears calcium from the cytosol. The time course of the clearance is slower than without PMCA block and the calcium baseline is not reached after 350 s (Figure 6B, black full line). This is a similar behavior as in the corresponding experiment with carboxyeosin [Figure 6D in Bautista et al. (12)]. However, it is not known whether the real CRAC allows for such inverse current under zero calcium conditions. The return of calcium to the baseline might also be supported by uptake of calcium by other organelles like mitochondria, which is not covered by the present model.

In the case of PMCA- and CRAC-block with La^3+^ a rather slow calcium clearance is observed in experiments which apply the same stimulation protocol [Figure 6C in Bautista et al. (12)]. *In silico* no clearance is observed at all. SERCA is blocked and positive *I*_IP3R_-currents are not allowed in the model, such that an uptake of calcium into the ER is excluded. A full block of PMCA and CRAC also excludes any expulsion of calcium out of the cell. Thus, the model suggests that the slow clearance of cytosolic calcium is either due to incomplete block of PMCA or to leakage currents.

### 3.5 Block of PMCA in an untreated TC

The model suggests that the role of CRAC for TC activation is mainly the maintenance of the integrity of the TCs during stimulation in the sense that it prevents the activated TC from running out of calcium. If we block PMCA *in silico* at the time of TCR stimulation (protocol as in Figure 3) in an otherwise untreated TC, the TC would be prevented of losing calcium. According to our interpretation of the role of CRAC we would expect that CRAC activity is strongly reduced in comparison to Figure 3.

**Figure 7:**
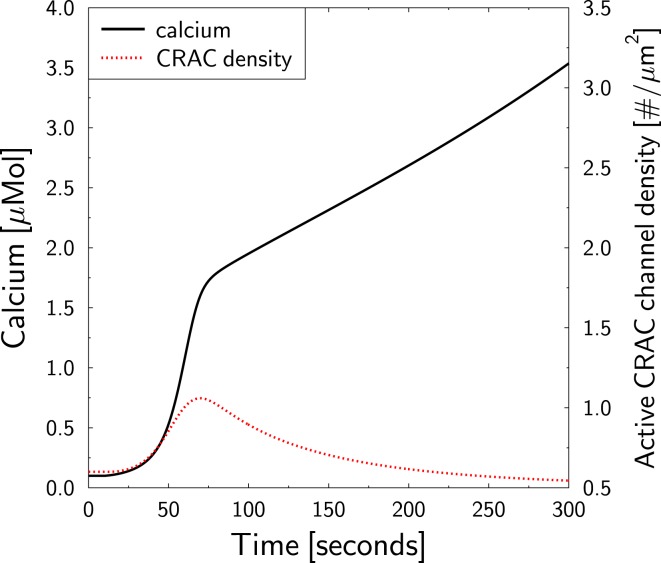
**Block of PMCA in untreated TCs**. The TCR stimulation protocol in Figure 3 is repeated. At the time of stimulation (*t* = 10 s), PMCA activity is blocked preventing calcium efflux from the cell. Cytosolic calcium (full black line, left axis) and the response of the CRAC density (dotted red line, right axis) are shown. The axis for the CRAC density was scaled as in Figure 3D for better comparison.

T lymphocytes receptor stimulation of PMCA-blocked TCs is predicted to induce a strong increase of cytosolic calcium (Figure 7, full black line). Thereby, the steady state CRAC current is not increased (as in Figure 3B, full black line) but reduced (not shown). However, the CRAC current is not reduced to zero such that the total block of PMCA infers a persistent net influx of calcium into the cell and, thus, to a persistent increase of cytosolic calcium. This would ultimately destroy a real TC. As SERCA activity is normal in this *in silico* experiment, calcium in the ER is only transiently reduced (not shown), leading to a transient and weak increase in the CRAC density (Figure 7, dotted red line). The amplitude is less than twofold instead of sixfold in Figure 3D.

This result supports the interpretation of the role of CRAC-channels *in silico* and may be tested in experiment. It further shows, that the calcium level in the ER may be used as an indicator for the overall calcium status of TCs.

## 4 Discussion

Within the presented investigation whole cell calcium dynamics were derived from models of single transmembrane ion-conducting proteins. The model successfully described a number of experimental settings and captured important characteristics of calcium dynamics upon TCR stimulation. In particular, the role of store-derived calcium-release versus CRAC activation was well represented. We conclude, that we have generated a modeling framework suitable for the quantitative analysis of calcium dynamics of TCs.

The value of using measured single transmembrane protein characteristics is twofold: at first, it substantially reduces the number of free parameters in an otherwise very complex model. All measured single protein properties were just implemented and not altered in the fine-tuning of the model. The reduced number of free parameters increases the predictive power of the model. Secondly, it can be considered as a multi-scale approach which links single proteins to whole cell behavior. This allows the analysis of the dynamics on the single protein level and its implications onto the cellular properties. The drawback of this approach is that not all parameters could be derived from TCs such that we had to assume that single transmembrane properties are universal, which is a correct assumption in many cases (30). Within this framework, cell-specific properties are controlled by the protein densities and the cell-specific properties, both determining the activity range of the respective proteins. However, the predictive power of the model would benefit from corresponding TC related data.

The model of Jurkat TC calcium dynamics as supported by the mathematical model starts from a TCR-derived increase in second messengers like IP3. This ultimately activates IP3R currents and induces an initial calcium current from the ER into the cytosol which triggers CICR. However, calcium uptake via SERCA activity equilibrates the calcium loss in the ER, which leads to a zero net flux on long-term – despite ongoing TCR stimulation. The ER calcium loss, according to the model, was the major contribution to the free cytosolic calcium peak. However, as PMCA activity leads to an overall loss of calcium in the cell, a compensation mechanism is required for a sustained elevation of free cytosolic calcium. The model suggests that the CRAC activity essentially contributes to this compensation mechanism. The initiation of CRAC currents by the depletion of ER calcium levels is in line with this interpretation. The model predicts, that a standard stimulation of TCs together with a block of PMCA activity would lead to a strong calcium rise together with a minor and transient increase of the CRAC density and on long-term to a reduced CRAC current (see Figure 7). This prediction may be tested in experiment in order to validate this interpretation of the role of CRAC-channels.

The emerging hypothesis that the role of CRAC is the stabilization of the TC calcium level, needs to be further strengthened by more detailed modeling work. In particular, early events after stimulation like NAADP generation (87) and subsequent RyR calcium currents from the ER are essential for the early calcium rise (11, 18) and will have to be included in the mathematical model for a proper time-resolved coverage of calcium dynamics. The need for additional mechanisms is also underpinned by the strong sensitivity of the model behavior to changes in the IP3 resting concentration *P*_0_ (Table 1). In the present simulation the long-term calcium plateau height mostly relies on the maximum possible CRAC activation by store-operated calcium depletion. For the long-lasting calcium rise, cADPR was proven relevant (16, 40) and has to be considered in the context of the present hypothesis on the role of CRAC. Also the transferability to human blood derived TCs, which exhibit a different geometry, has to be assessed.

As the PMCA block experiment at zero external calcium has shown, it might be important to include leakage currents into the model. However, it should be noted that the postulated role of delayed PMCA activity for the calcium overshoot after TCR stimulation (12) could only partially be confirmed by the mathematical model. With fast activation of PMCA, an overshoot was still observed in our model, and the overshoot could only be suppressed by lack of IP3R inactivation. It should be noted that this result might rely on the Mak–McBride–Foskett model (37), which was used for IP3R dynamics and which exhibits inactivation at rather low IP3. The result might differ if the TC calcium dynamics would be based on the DeYoung–Keizer model for IP3R activity (65).

The proposed model has limitations in its range of applicability. For example, the usage of the Nernst-equation for the chemical gradient and Ohm’s law for the current-voltage relationship of ion-conducting pores is justified only in narrow limits. Experiments with zero external calcium drive the model to the very limits of this range of applicability which was circumvented here using a phenomenological approximation. The model may be reformulated in terms of the Fokker-Planck-equation in order to describe ion transport through the pores in more detail. Furthermore, it is known that calcium entrance points lead to spatially inhomogeneous calcium dynamics (22) which are not covered by the present space-averaged model. The value of the present approach lies in the surprising result that quantitative characteristics of single transmembrane proteins are sufficient to determine the cell behavior in the framework of an ordinary-differential-equation based model. The model has proven its predictive power, as it was fitted to data of one experiment in Figure 3 and could be used to predict and explain further data of calcium dynamics generated under other experimental conditions in Figures 4 and 6. It is planned to elaborate the potential and the limits of the model predictions by application to further experimental settings.

## Conflict of Interest Statement

The authors declare that the research was conducted in the absence of any commercial or financial relationships that could be construed as a potential conflict of interest.
